# Supplementary material for: DMRforPairs: identifying Differentially Methylated Regions between unique samples using array based methylation profiles
Source: BMC Bioinformatics. 2014 May 15;15:141. doi: 10.1186/1471-2105-15-141 (PMC4046028; doi:10.1186/1471-2105-15-141)
Supplement: Additional file 2 — DMRforPairs output for the comparison of A431-MCF7 and NA17018-NA17105. Please start from the HTML files in each folder. Available via the BMC Bioinformatics website. [file 1471-2105-15-141-S2.zip › 1394847754114233_MOESM2_ESM/A431_MCF7/figures/7577.pdf]

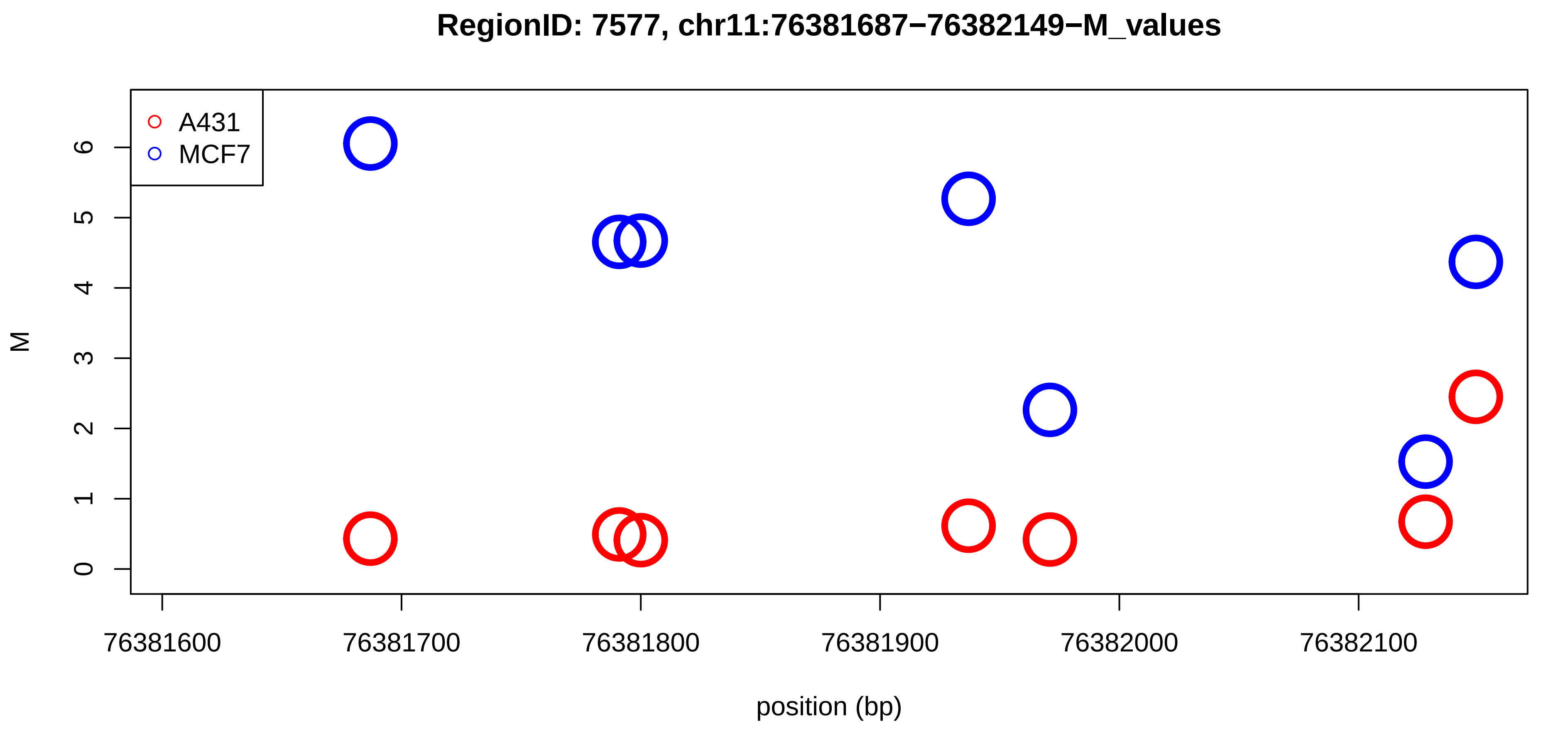
RegionID: 7577, chr11:76381687−76382149−M_values
A431
MCF7
M
0
1
2
3
4
5
6
76381600
76381700
76381800
76381900
76382000
76382100
position (bp)

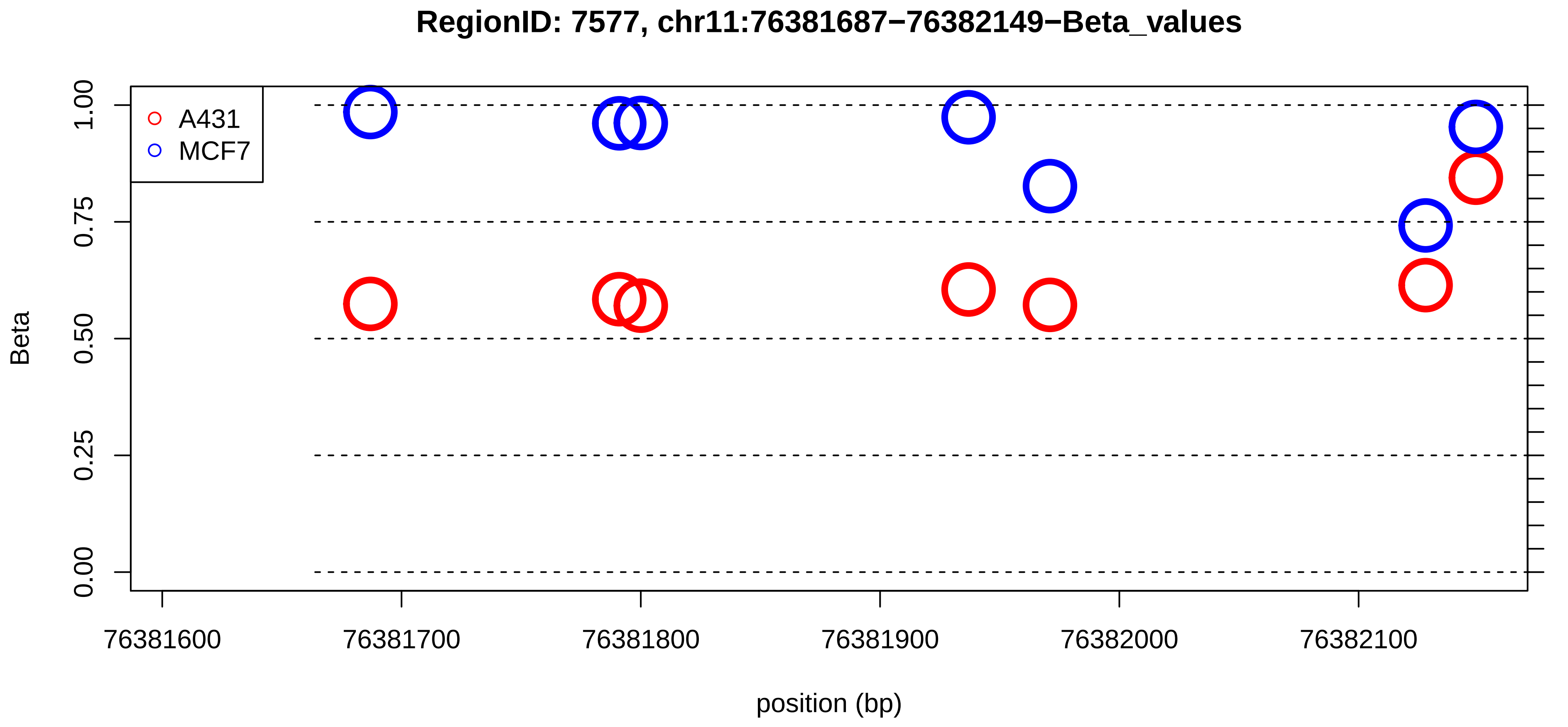
RegionID: 7577, chr11:76381687−76382149−Beta_values
A431
MCF7
Beta
0.00
0.25
0.50
0.75
1.00
76381600
76381700
76381800
76381900
76382000
76382100
position (bp)

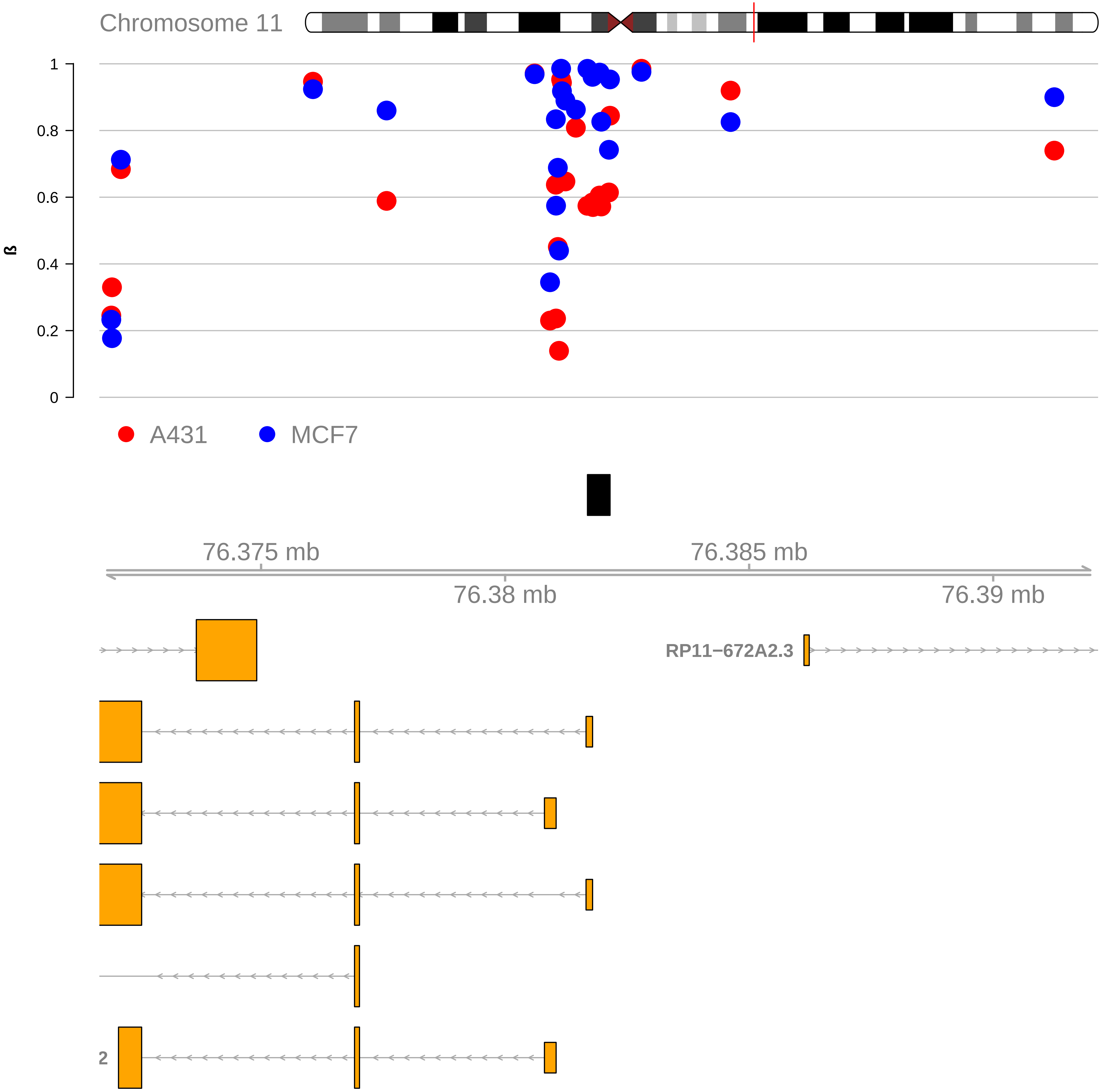

Chromosome 11
1
0.8
0.6
0.4
0.2
0
ß
A431
MCF7
76.375 mb
76.385 mb
76.38 mb
76.39 mb
RP11−672A2.3
2
